# Feasibility, acceptability, and functional outcomes of a home-based exergaming telerehabilitation program for adults with functional disabilities: a multi-center single-arm pilot study

**DOI:** 10.1186/s12984-026-02055-x

**Published:** 2026-06-16

**Authors:** Ravi Shankar, Ruth Choo Li Ong, Emily Yee, Ramesh Duraisamy, Benedict Chung Yin Ho, Rena Sim, Christopher Wee Keong Kuah, Poo Lee Ong

**Affiliations:** 1 Clinical Research and Innovation Office, National Healthcare Group, Tan Tock Seng Hospital, 11 Jalan Tan Tock Seng, 308433 Singapore, Singapore; 2 Institute of Rehabilitation Excellence (IREx), Tan Tock Seng Hospital Rehabilitation Centre, Singapore, Singapore; 3 Handicaps Welfare Association (HWA), Singapore, Singapore; 4 Home Nursing Foundation (HNF), Singapore, Singapore; 5 Ren Ci Hospital, Singapore, Singapore

**Keywords:** Telerehabilitation, Exergaming, Gamification, Community rehabilitation, Balance training, Functional disabilities, Feasibility study

## Abstract

**Background:**

Community-based rehabilitation services face significant challenges including limited access, poor exercise adherence, and resource constraints. Telerehabilitation using gamified exergaming platforms offers a promising solution to extend rehabilitation services into home settings while maintaining patient engagement.

**Objective:**

This multi-center single-arm pilot study evaluated the feasibility, acceptability, and preliminary efficacy signals of a caregiver-supported, home-based telerehabilitation program comprising 30 days of active home use within an 18-week protocol, using the EvolvRehab gamified exergaming platform among community-dwelling adults with functional limitations who were receiving outpatient rehabilitation in Singapore.

**Methods:**

Ten participants (mean age: 64.6 years, SD 8.5, range 48–78) were recruited from three community rehabilitation sites: Handicaps Welfare Association Day Rehabilitation Centre, Home Nursing Foundation, and Ren Ci Hospital Day Rehabilitation Centre. Participants underwent an 18-week protocol comprising onboarding, a 30-day period of active home-based telerehabilitation with therapist-prescribed daily exercises and remote monitoring, and follow-up. A primary caregiver was present during all home sessions to assist with device setup, supervise exercise, and support fall prevention. Functional outcomes were assessed at baseline (Week 0), post-intervention (Week 8), and follow-up (Week 18) using the Modified Barthel Index (MBI), Short Physical Performance Battery (SPPB), Berg Balance Scale (BBS), grip strength, and EQ-5D-5 L. System usability and patient-reported outcomes were evaluated using the System Usability Scale (SUS) and a non-validated custom questionnaire, the latter analysed as exploratory data.

**Results:**

Mean exercise compliance was 80.6%, with participants exercising an average of 23.97 min daily. Within-group improvements from baseline to Week 18 follow-up were observed in MBI (75.8 to 81.2, *p* = 0.019), BBS (33.3 to 38.0, *p* = 0.037), and right-hand grip strength (15.1 to 18.0 kg, *p* = 0.005). SPPB improved at the post-intervention assessment (5.2 to 6.2, *p* = 0.015), but this change was not sustained at the Week 18 follow-up relative to baseline (6.4, *p* = 0.104). EQ-5D-5 L descriptive sum scores decreased (indicating reduced symptom burden) from 11.6 at baseline to 8.5 at Week 8 (*p* = 0.002; FDR-adjusted *p* = 0.048) and 8.7 at Week 18; EQ-VAS scores increased from 66.9 at baseline to 80.9 at Week 18 (*p* = 0.022). As an uncontrolled single-arm study, these findings represent preliminary efficacy signals rather than evidence of effectiveness, and require confirmation in controlled trials. The mean SUS score was 67.75, indicating marginal acceptability. Patient-reported outcomes showed high engagement with the gamified exercises, though concerns about cost were noted. No falls or adverse events were reported in this selected sample under caregiver-supported conditions.

**Conclusions:**

Gamified caregiver-supported telerehabilitation using the EvolvRehab platform appeared feasible and acceptable among community-dwelling adults with functional limitations, and was associated with within-group functional outcome signals. High compliance rates and the absence of adverse events under caregiver-supported conditions support the conduct of larger controlled trials, though the uncontrolled single-arm design precludes conclusions about effectiveness, and usability refinements and cost considerations require attention for scalability.

## Introduction

 Rehabilitation services play a critical role in restoring functional independence and quality of life for individuals with disabilities. However, community-based rehabilitation faces persistent challenges globally, including geographic barriers, limited healthcare workforce, transportation difficulties, and poor adherence to exercise programs [1, 2]. In Singapore, these challenges are compounded by an aging population and increasing demand for rehabilitation services. Local studies have documented concerning patterns of rehabilitation discontinuation, with adherence rates at day rehabilitation centers estimated at only 28% at one year post-discharge [3], and rehabilitation attendance rates of approximately 20% at three months following hospitalization [4].

The barriers to community rehabilitation adherence are multifactorial, encompassing functional limitations, social constraints, financial concerns, and perceptual factors [5]. Furthermore, home-based self-exercise programs, while commonly prescribed, suffer from non-adherence rates as high as 70%, influenced by poor self-motivation, low self-efficacy, and limited social support [6]. These challenges underscore the need for innovative service delivery models that can overcome traditional barriers while maintaining therapeutic effectiveness and patient engagement.

Telerehabilitation has emerged as a promising approach to address these challenges by leveraging information and communication technologies to deliver rehabilitation services remotely [7, 8]. Systematic reviews have demonstrated that telerehabilitation can achieve outcomes comparable to conventional face-to-face rehabilitation across various conditions including stroke, musculoskeletal disorders, and chronic diseases [9, 10]. Recent evidence suggests that telerehabilitation exhibits a generally safe profile with most adverse events being rare, non-serious, or unrelated to the intervention protocol [2]. The COVID-19 pandemic accelerated the adoption of telerehabilitation globally, demonstrating its viability as an alternative or complement to traditional in-person services.

Within telerehabilitation, exergaming, the use of video games that involve physical exercise has gained attention to enhance motivation and adherence through gamification elements. Exergaming combines therapeutic exercises with interactive gaming mechanics, providing real-time feedback, goal-setting features, and engaging visual interfaces that can transform monotonous exercises into enjoyable activities [11]. Studies have shown that exergaming can improve balance, physical function, and fall prevention outcomes in older adults, with high doses (above 134 min per week) appearing particularly beneficial [12, 13].

The EvolvRehab system is a CE-certified non-immersive virtual rehabilitation platform developed by Evolv, comprising a suite of therapy modules for upper and lower extremity rehabilitation. The system utilizes motion capture technology to track patient movements during gamified exercises, with capabilities for synchronous tele-monitoring, training scheduling, and progress monitoring via cloud connectivity. Previous research has demonstrated the system’s potential for delivering balance and fall prevention programs to older adults in aged care settings [12].

Despite growing evidence supporting telerehabilitation and exergaming, implementation in community settings remains limited, particularly in Asian contexts. Questions persist regarding acceptability among diverse patient populations, practical implementation challenges, and sustainability of outcomes. This multi-center single-arm pilot study aimed to evaluate the feasibility, acceptability, and preliminary efficacy signals of a caregiver-supported, home-based telerehabilitation program (comprising 30 days of active home use within an 18-week protocol) using the EvolvRehab exergaming platform among community-dwelling adults with functional limitations receiving outpatient rehabilitation in Singapore.

## Methods

### Study design

This prospective, multi-center, single-arm pilot clinical feasibility trial evaluated the implementation of home-based telerehabilitation through deployment of the EvolvRehab system using a single-group pre-post design with no control group. Reporting follows the CONSORT 2010 extension for pilot and feasibility trials. The study was approved by the National Healthcare Group Domain Specific Review Board (NHG DSRB Reference: 2023/00184) and was conducted in accordance with the Declaration of Helsinki. All participants provided written informed consent prior to enrollment. The study was funded by the Ng Teng Fong Healthcare Innovation Programme (NTF HIP). A priori feasibility progression thresholds were specified as follows: a recruitment rate sufficient to enrol the target sample within the funding period, a retention rate of at least 70%, and a mean exercise compliance of at least 70% of prescribed days. These thresholds were used to judge the feasibility of progressing to a larger controlled trial.

### Setting and participants

Participants were recruited from three community rehabilitation sites in Singapore: Handicaps Welfare Association (HWA) Day Rehabilitation Centre, Home Nursing Foundation (HNF), and Ren Ci Community Hospital Day Rehabilitation Centre. Recruitment occurred between 2023 and 2024, with coordination provided by Tan Tock Seng Hospital Rehabilitation Centre. The intended target population was community-dwelling adults with functional limitations affecting mobility, balance, or activities of daily living, of sufficient severity to require ongoing outpatient rehabilitation but not so severe as to preclude participation in a home-based exergaming program. For the purpose of participant selection, a functional limitation was operationally defined as an impairment in sitting, standing, walking, or balance that the treating therapist judged to warrant continued structured rehabilitation, in the context of a neurological or musculoskeletal diagnosis. Eligible participants were identified by the rehabilitation therapists at each site from their active outpatient caseload and were screened against the eligibility criteria below. The resulting sample was characterised by relatively low baseline functional scores and a meaningful risk of falls rather than by general independence, and this should be considered when interpreting the findings.

*Inclusion criteria* [1] Currently receiving outpatient rehabilitation services from one of the participating sites; [2] Age 21–90 years; [3] Able to perform sitting, standing, and/or walking tasks with no more than minimal assistance (caregiver provides up to 25% physical support); [4] Able to follow 1–2 step verbal commands consistently; [5] Capacity to understand and provide informed consent; [6] Primary caregiver available to be present during all sessions.

*Exclusion criteria* [1] Severe cognitive, visual, perceptual, or emotional-behavioral issues precluding participation; [2] Moderate to severe pain (Visual Analogue Scale > 5); [3] History of seizure episodes; [4] Unstable medical conditions or anticipated life expectancy < 1 year; [5] Pregnancy or lactation; [6] Requires assistance of two people for exercise or high fall risk without competent caregiver.

### Intervention

The EvolvRehab system is a non-immersive virtual rehabilitation platform utilizing motion capture technology to deliver gamified therapeutic exercises. The system consists of a compact device suitable for home deployment, capable of tracking full-body movements and providing real-time feedback during exercise sessions. Participants interact with game-based therapy modules designed for upper extremity, lower extremity, and trunk rehabilitation.

**Fig. 1:**
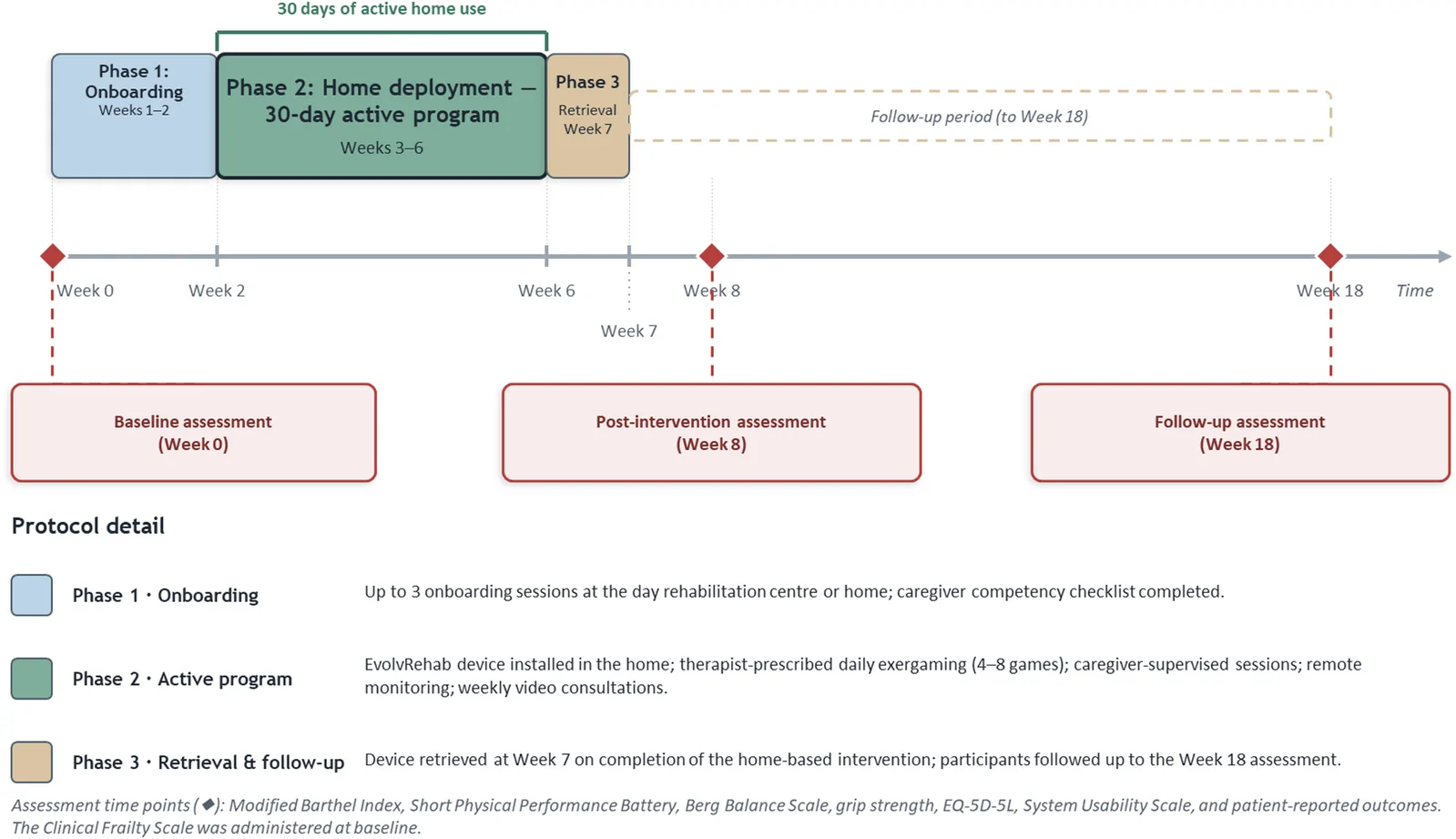
The study timeline

The intervention protocol comprised three phases over 18 weeks. The program was delivered as caregiver-supported, caregiver-supervised telerehabilitation rather than as fully independent home telerehabilitation. A primary caregiver was required to be present throughout every home session and was responsible for device setup, supervision of the participant during exercise, prompting and pacing, fall prevention, and provision of physical assistance when needed. During Weeks 1–2, participants completed up to three onboarding sessions at their respective day rehabilitation centers or homes to familiarize them with the system and ensure caregiver competency. Caregivers completed a competency checklist demonstrating ability to assist with device setup, exercise supervision, and fall management. During Weeks 3–6, the EvolvRehab device was installed in participants’ homes for a 30-day telerehabilitation program. Therapists prescribed individualized daily exercise programs consisting of 4–8 games targeting balance, strength, and coordination. Participants were encouraged to exercise daily, with progress monitored remotely via the cloud-based platform. Exercise duration was not prescriptively fixed per session; therapists assessed and prescribed exercises according to each participant’s tolerance level, with a maximum limit of 60 min per day to prevent injury. Weekly video consultations provided opportunities for program adjustments and feedback. At Week 7, devices were retrieved upon completion of the home-based intervention. Several processes were used to ensure participant safety during the home-based phase. Participants exercised only in the presence of a competency-checked caregiver; exercises were prescribed and progressed according to therapist-assessed tolerance; and participants or caregivers were instructed to stop a session and contact the study team in the event of pain, dizziness, undue fatigue, or any concern about safety. Weekly video consultations were used to review tolerance and adjust or discontinue exercises as required. Technical problems were reported to the study team and resolved remotely or through a home visit, with the program paused for that participant until the issue was resolved. To clarify the sequence of study activities, the protocol proceeded as follows: onboarding and caregiver competency training during Weeks 1–2; home deployment of the device and the 30-day active telerehabilitation program during Weeks 3–6; device retrieval at Week 7; post-intervention assessment at Week 8; and follow-up assessment at Week 18. The 30-day period of active home use therefore sat within the longer 18-week protocol window. The study timeline is summarised in Fig. 1.

### Outcome measures

Outcomes were assessed at three time points: baseline (Week 0), post-intervention (Week 8), and follow-up (Week 18). All assessments were conducted by trained therapists at the respective rehabilitation sites.

*Primary outcomes (feasibility and acceptability)* Exercise compliance was defined as the percentage of days over the 30-day period during which the participant exercised for more than 20 min. The System Usability Scale (SUS), a validated 10-item questionnaire yielding scores from 0 to 100, assessed perceived system usability. Scores above 68 indicate above-average usability, with scores 50–70 considered marginally acceptable [14]. Patient-reported outcome measures (PROMs) evaluated engagement, perceived benefit, and willingness to continue using the system. PROMs were collected using a custom questionnaire developed by the study team for this pilot. This instrument has not undergone formal validation, and no data on item development, content validity, or reliability are available; its results are therefore reported as exploratory and are not used to support definitive acceptability claims. Safety outcomes were monitored throughout the home-based phase. Adverse events were defined as any untoward medical occurrence during the study period; falls were defined as any unintentional coming to rest on the ground or a lower level; near-falls were defined as a loss of balance that did not result in a fall; and pain exacerbation and sessions interrupted due to fatigue were also recorded. Caregivers and participants reported these events to the study team, and events were additionally reviewed during weekly video consultations.

*Secondary outcomes (clinical outcomes)* The Modified Barthel Index (MBI) assessed activities of daily living on a 0-100 scale. The Short Physical Performance Battery (SPPB) evaluated lower extremity function through balance, gait speed, and sit-to-stand tests (range 0–12). The Berg Balance Scale (BBS) assessed static and dynamic balance through 14 items (range 0–56). Grip strength was measured using a handheld dynamometer, with three trials averaged for each hand. The EQ-5D-5 L assessed health-related quality of life across five dimensions. EQ-5D-5 L responses were summarised as the descriptive system sum score and the EQ visual analogue scale (EQ-VAS); a utility index was not derived for this pilot. The Clinical Frailty Scale (CFS) was administered at baseline to characterise frailty and to define the frailty subgroups used in the analysis; the CFS was scored by the assessing therapist according to the standard nine-point scale.

### Statistical analysis

Descriptive statistics summarized participant characteristics, compliance metrics, and outcome measures at each time point. Continuous variables were expressed as means with standard deviations or, where distributions were skewed, as medians with interquartile ranges, and categorical variables as frequencies and percentages. Given the small sample size (*N* = 10), the distribution of each outcome was examined using the Shapiro-Wilk test before analysis. Where normality was supported, the paired t-test was used to compare outcome measures between time points; otherwise the Wilcoxon signed-rank test was used. The specific test applied to each comparison is indicated in the corresponding table footnote. For each within-group comparison, the mean change with its 95% confidence interval was reported, and an effect size was calculated (Hedges’ g for parametric comparisons and the matched-pairs rank-biserial correlation for non-parametric comparisons) to inform the design of future trials. Statistical significance was set at *p* < 0.05. Because ten pairwise comparisons were made across outcomes and time points, the Benjamini-Hochberg false discovery rate procedure was applied to control for multiple testing, and both unadjusted and adjusted p-values were reported. Likert-type items from the patient-reported outcome questionnaire were treated as ordinal data and summarised using medians, interquartile ranges, and full response distributions rather than means. Given the pilot nature of the study, formal power calculations were not performed; the target sample of 20 participants was based on funding constraints and anticipated dropout rates of 15–20%.

## Results

### Participant characteristics

**Fig. 2:**
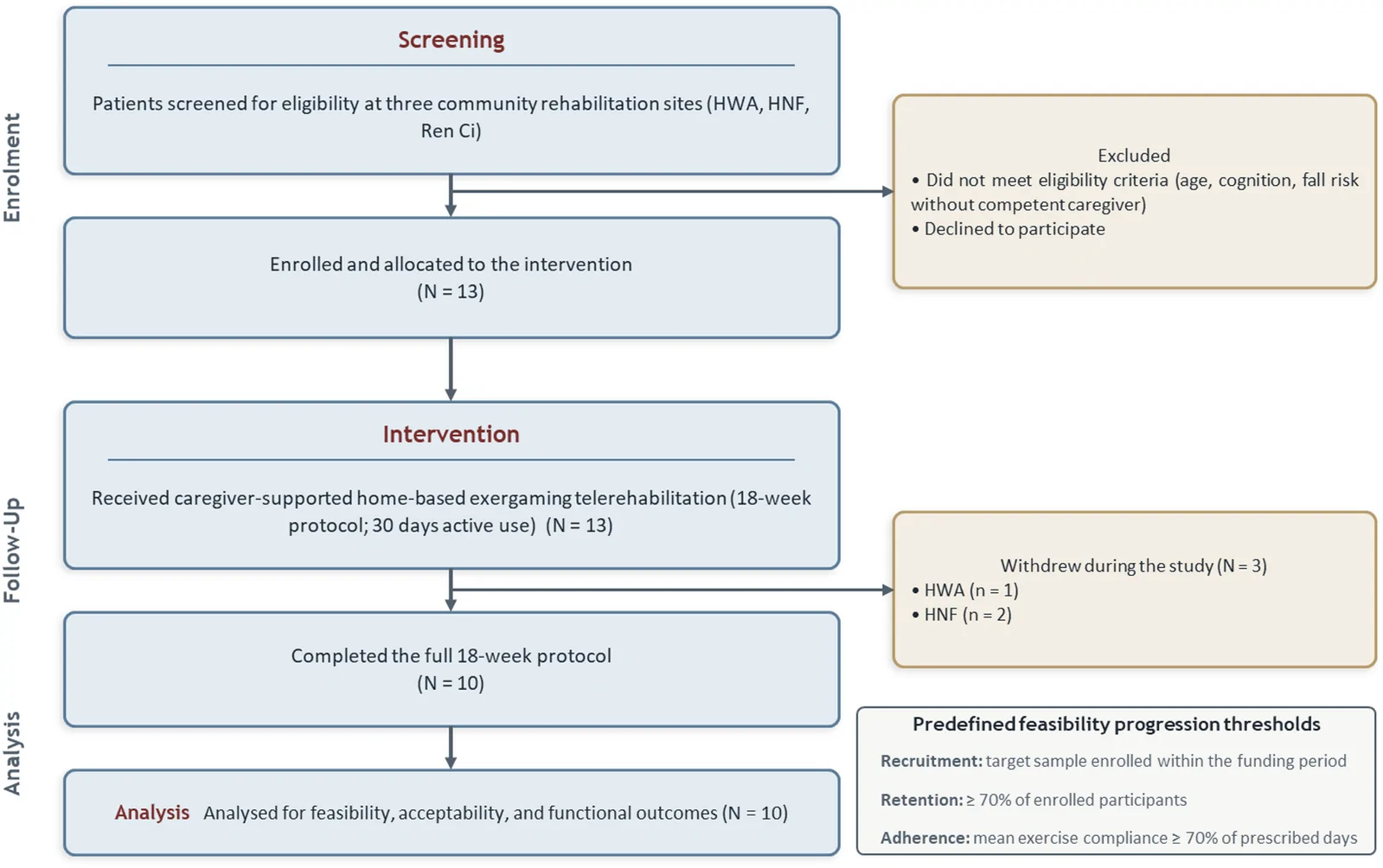
CONSORT 2010 flow diagram of the study participants. Participant flow through screening, enrolment, intervention, follow-up, and analysis. As a single-arm pilot study, there was no randomisation or control group

Thirteen participants were enrolled across the three sites: seven from HWA, five from HNF, and one from Ren Ci. Three participants withdrew during the study (one from HWA and two from HNF), resulting in ten participants completing the full protocol and being included in the analysis. The flow of participants through the study (screened, eligible, enrolled [*N* = 13], withdrew [*N* = 3], completed [*N* = 10], and analysed [*N* = 10]) is summarised in the CONSORT 2010 pilot and feasibility extension flow diagram (Fig. 2). Table 1 presents participant demographics and clinical characteristics.

**Table 1:** Participant demographics and clinical characteristics (*N* = 10)

Characteristic	Value
Age, mean ± SD [range] years	64.6 ± 8.5 [48–78]
Gender, n (%)	
Male	4 (40%)
Female	6 (60%)
Race/Ethnicity, n (%)	
Chinese	7 (70%)
Malay	2 (20%)
Indian	1 (10%)
Primary diagnosis, n (%)	
Neurological conditions	4 (40%)
Musculoskeletal conditions	4 (40%)
Other (deconditioning following medical hospitalisation and cardiopulmonary conditions)	2 (20%)
Clinical Frailty Scale, n (%)	
Mild frailty	5 (50%)
Moderate frailty	2 (20%)
Severe frailty	3 (30%)

The mean age of completers was 64.6 years (SD 8.5, range 48–78), with 60% female participants. The ethnic composition reflected Singapore’s demographics, with Chinese (70%), Malay (20%), and Indian (10%) representation. Primary diagnoses included neurological conditions (40%), musculoskeletal conditions (40%), and other conditions (20%); the “other” category comprised participants with deconditioning following medical hospitalisation and with cardiopulmonary conditions. Participants were stratified into three frailty subgroups based on the Clinical Frailty Scale (CFS), which was administered at baseline as described in the Outcome Measures section: mild frailty, moderate frailty, and severe frailty; the distribution of participants across these subgroups is shown in Table 1.

### Feasibility and compliance

Exercise compliance across all participants was high, with a mean compliance rate of 80.6% (range 53.3–96.7%). Nine of ten participants (90%) achieved compliance rates exceeding 70%. Participants completed a mean of 38.6 total sessions over the 30-day intervention period, with mean daily exercise duration of 23.97 min. For two participants (HNF001 and RC001), the total session count recorded by the platform (74 and 79 respectively) reflects the number of discrete game-play logs rather than the number of distinct exercise days, because the platform recorded each game module within a single daily session as a separate entry for these participants. The number of distinct days on which exercise was performed remained within the 30-day window and is consistent with the compliance percentages reported. Table 2 presents individual compliance data.

**Table 2:** Exercise compliance and usage metrics by participant

Participant ID	Compliance (%)	Total sessions (game-play logs)	Mean duration (min/day)	Games prescribed
HWA002	90.0	35	27.5	8
HWA003	93.3	28	22.3	8
HWA004	86.7	27	28.4	7–8
HWA005	70.0	21	30.0	7–8
HWA006	76.0	35	18.1	4–5
HWA007	53.3	22	13.6	6
HNF001	96.7	74	28.2	4–7
HNF002	70.0	35	30.3	7
HNF003	90.0	30	13.6	8
RC001	80.0	79	27.8	4
Mean ± SD	80.6 ± 13.9	38.6 ± 19.8	24.0 ± 6.6	-

“Total Sessions” denotes the number of game-play logs recorded by the platform. For HNF001 and RC001, each game module completed within a single daily session was logged as a separate entry, so the figures shown (74 and 79) exceed the number of distinct exercise days. Compliance (%) is based on the number of distinct days on which the participant exercised for more than 20 min, within the 30-day intervention window

### Clinical outcomes

Table 3 presents the functional outcome measures across all assessment time points. Within-group changes from baseline were observed in several domains, with mean changes, 95% confidence intervals, effect sizes, and both unadjusted and false discovery rate (FDR) adjusted p-values reported. As this was an uncontrolled single-arm study, these changes are reported as functional outcome signals and cannot be attributed to the intervention.

**Table 3:** Functional outcomes at baseline, post-intervention, and follow-up

Outcome measure	Baseline (Week 0)	Post-intervention (Week 8)	Follow-up (Week 18)
MBI	75.8 ± 27.1	80.4 (*p* = 0.023)* ± 26.6; Δ + 4.6 (95% CI + 0.80, + 8.40); Hedges’ g = + 0.16; paired t, FDR-adjusted *p* = 0.060	81.2 (*p* = 0.019)* ± 26.2; Δ + 5.4 (95% CI + 1.12, + 9.68); Hedges’ g = + 0.19; paired t, FDR-adjusted *p* = 0.060
SPPB	5.2 ± 4.3	6.2 (*p* = 0.015)* ± 4.7; Δ + 1.0 (95% CI + 0.25, + 1.75); Hedges’ g = + 0.21; paired t, FDR-adjusted *p* = 0.060	6.4 (*p* = 0.104) ± 4.6; Δ + 1.2 (95% CI -0.30, + 2.70); Hedges’ g = + 0.26; paired t, FDR-adjusted *p* = 0.168; not statistically significant relative to baseline
BBS	33.3 ± 18.4	36.4 (*p* = 0.103) ± 20.9; Δ + 3.1 (95% CI -0.77, + 6.97); Hedges’ g = + 0.15; paired t, FDR-adjusted *p* = 0.168	38.0 (*p* = 0.037)* ± 20.0; Δ + 4.7 (95% CI + 0.35, + 9.05); Hedges’ g = + 0.23; paired t, FDR-adjusted *p* = 0.086
Right grip strength (kg)	15.1 ± 6.8	16.7 (*p* = 0.057) ± 7.3; Δ + 1.6 (95% CI -0.06, + 3.33); Hedges’ g = + 0.22; paired t, FDR-adjusted *p* = 0.109	18.0 (*p* = 0.005)* ± 7.3; Δ + 2.9 (95% CI + 1.11, + 4.66); Hedges’ g = + 0.39; paired t, FDR-adjusted *p* = 0.053
Left grip strength (kg)	16.2 ± 10.5	17.2 (*p* = 0.238) ± 10.2; Δ + 1.0 (95% CI + 0.32, + 1.78); Hedges’ g = + 0.10; paired t, FDR-adjusted *p* = 0.060	17.6 (*p* = 0.238) ± 8.1; Δ + 1.4 (95% CI -1.10, + 3.89); Hedges’ g = + 0.14; paired t, FDR-adjusted *p* = 0.294

Values are mean ± standard deviation. For each post-baseline time point, the mean change from baseline (Δ) is reported with its 95% confidence interval, the effect size (Hedges’ g for paired t-tests; matched-pairs rank-biserial correlation for Wilcoxon signed-rank tests), and the false discovery rate (FDR) adjusted p-value alongside the unadjusted p-value. All comparisons are relative to baseline. The Shapiro-Wilk test was used to assess normality, and the corresponding parametric or non-parametric test was applied for each comparison. *Unadjusted *p* < 0.05. MBI = Modified Barthel Index; SPPB = Short Physical Performance Battery; BBS = Berg Balance Scale

The Modified Barthel Index showed within-group increases from baseline (75.8) to both post-intervention (80.4, unadjusted *p* = 0.023) and follow-up (81.2, unadjusted *p* = 0.019), with a further increase between Week 8 and Week 18 (unadjusted *p* = 0.037). The corresponding mean changes, confidence intervals, effect sizes, and FDR-adjusted p-values are reported in Table 3. As the study had no control group, these changes are described as functional outcome signals associated with the program rather than as effects attributable to it. Individual participant analysis revealed improvements across all three frailty subgroups (mild, moderate, and severe frailty) over the 18-week period.

The Short Physical Performance Battery increased from baseline to the post-intervention assessment (5.2 to 6.2, unadjusted *p* = 0.015). For reference, Perera et al. (2006) reported a minimal clinically important difference (MCID) of approximately 1.0 point for the SPPB [20]; the observed post-intervention change of 1.0 point is of a comparable magnitude. The Week 18 value (6.4) was numerically higher than baseline, but the comparison to baseline was not statistically significant (unadjusted *p* = 0.104), indicating that the post-intervention improvement was not sustained at follow-up. This pattern should be interpreted with caution given the small sample size and the absence of a control group.

The Berg Balance Scale showed a progressive within-group increase across time points, with the baseline to follow-up change (33.3 to 38.0, unadjusted *p* = 0.037) reaching statistical significance before adjustment for multiple comparisons. The 4.7-point improvement approaches the MCID of 5 points reported for stroke patients requiring walking assistance [15]. Subgroup analysis revealed that participants in the mildly frail group showed consistent improvements, while those in the pre-frail group at baseline demonstrated ceiling effects.

**Fig. 3:**
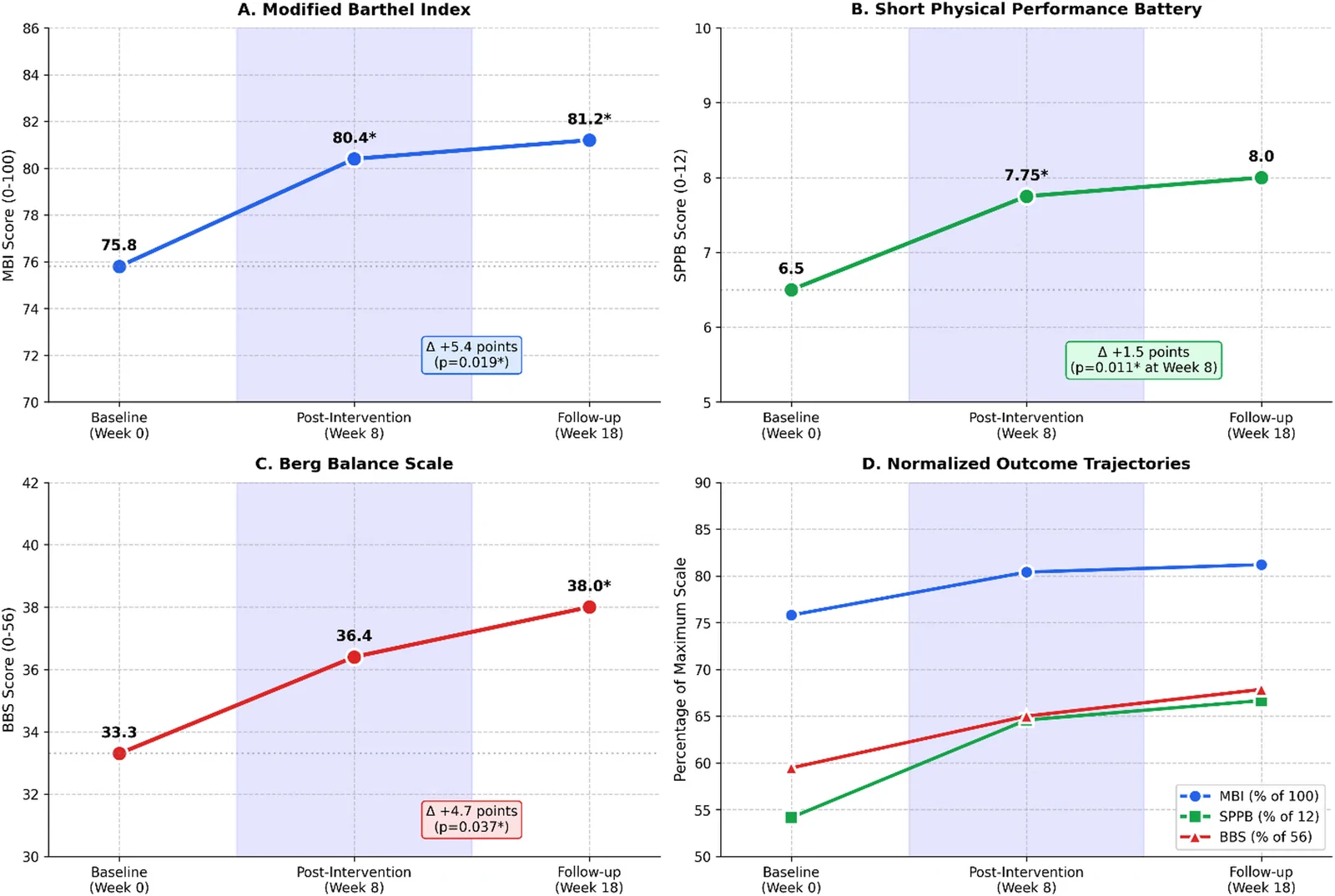
Graphs showing changes in MBI, SPPB, BBS, and normalized outcome trajectories scores across the three assessment time points (Week 0, Week 8, Week 18)

Right-hand grip strength increased within-group from baseline to follow-up (15.1 to 18.0 kg, unadjusted *p* = 0.005), with increases observed across all frailty subgroups. Left-hand grip strength showed smaller within-group changes that did not reach statistical significance. As with the other outcomes, these are uncontrolled within-group changes and cannot be attributed to the intervention (Fig. 3).

### Quality of life

EQ-5D-5 L responses were summarised as the descriptive system sum score (range 5–25; lower is better) and as the EQ visual analogue scale (EQ-VAS; range 0-100; higher is better). A utility index was not derived for this pilot. The EQ-VAS and descriptive sum score at baseline, Week 8, and Week 18 are presented in Table 4. Within-group changes were in the direction of improvement: the descriptive sum score decreased from 11.6 (SD 4.0) at baseline to 8.5 (SD 4.2) at Week 8 and 8.7 (SD 3.6) at Week 18, with a Week-0-to-Week-8 change of -3.10 (95% CI -4.77, -1.43; Hedges’ g = -0.72; paired t, FDR-adjusted *p* = 0.048). The EQ-VAS increased from 66.9 (SD 16.3) at baseline to 80.9 (SD 18.4) at Week 18, with a Week-0-to-Week-18 change of + 14.0 (95% CI + 2.51, + 25.49; Hedges’ g = + 0.77; paired t, FDR-adjusted *p* = 0.060). Because the study was uncontrolled, these changes are reported descriptively and are not interpreted as effects of the intervention.

**Table 4:** EQ-5D-5 L EQ-VAS and descriptive sum score at each time point (*N* = 10)

EQ-5D-5 L measure	Baseline (Week 0)	Post-Intervention (Week 8)	Follow-up (Week 18)
EQ-VAS, mean ± SD	66.9 ± 16.3	74.0 ± 17.8	80.9 ± 18.4
Descriptive sum score, mean ± SD	11.6 ± 4.0	8.5 ± 4.2	8.7 ± 3.6

EQ-VAS = EQ visual analogue scale (0-100); higher values indicate better self-rated health. The descriptive sum score is the unweighted sum of the five dimension levels (range 5–25); lower values indicate fewer reported problems. The EQ-5D-5 L utility index using the Singapore value set was not derived for this pilot; only the descriptive responses and EQ-VAS were collected

### System usability and patient-reported outcomes

The mean System Usability Scale score was 67.75 (range 42.5–80), placing the system in the “marginal” acceptability range. Five of ten participants (50%) provided scores of 70 or above, indicating good usability, while four participants (40%) found the system difficult to use, and one participant felt it was not a good fit for their needs. Table 5 presents the patient-reported outcome measures, which were collected using a non-validated custom questionnaire and are therefore reported as exploratory findings only.

**Table 5:** Patient-reported outcome measures from a non-validated custom questionnaire (exploratory) (*N* = 10)

Statement	Mean score (1–5) and Median [IQR]
The games were engaging	4.4; median 4 [IQR 4–5]
The games challenged me appropriately	4.5; median 4.5 [IQR 4–5]
I enjoyed playing the games and looked forward to sessions	3.9; median 4 [IQR 4–4]
I would be willing to pay for this system at face-to-face session cost	2.1; median 2 [IQR 2–3]
My overall health and physical function has improved	4.1; median 4 [IQR 4–4]
I am more willing to continue exercising by myself	3.9; median 3.5 [IQR 3–5]
This system helped me develop a regular exercise habit	3.9; median 3.5 [IQR 3–5]

Items were scored from 1 (Strongly Disagree) to 5 (Strongly Agree). Because these are ordinal Likert-type items, the median and interquartile range (IQR) are reported as the primary summary, with the mean retained for descriptive continuity. The questionnaire was developed by the study team for this pilot and has not been validated; no data on item development, content validity, or reliability are available. These results are therefore exploratory and do not by themselves support acceptability claims

Participants rated the games highly for engagement (mean 4.4) and appropriate challenge level (mean 4.5). Most participants perceived improvement in their physical function (mean 4.1) and expressed interest in continuing exercise (mean 3.9). However, willingness to pay for the system at the cost of face-to-face sessions was low (mean 2.1), with seven participants (70%) disagreeing or strongly disagreeing with this statement. These patient-reported ratings were obtained using a non-validated custom questionnaire and are reported here as exploratory observations only; the corresponding medians and interquartile ranges are presented in Table 5 once finalised. They are not used to support formal claims about acceptability, which rest instead on the objective compliance and retention data and the validated System Usability Scale.

### Safety

No adverse events, falls, near-falls, or episodes of pain exacerbation were reported throughout the study, and no sessions were recorded as interrupted due to fatigue. There were no falls, injuries, or medical complications related to the telerehabilitation intervention. This favourable safety profile should be interpreted in the context of a small, selected sample studied under caregiver-supported conditions: a competency-checked primary caregiver was present during every home session, exercises were prescribed and progressed according to therapist-assessed tolerance, weekly video consultations were used to review tolerance and adjust the program, and participants at high fall risk without an adequate caregiver were excluded. The findings therefore indicate that the program can be delivered safely under these specific conditions rather than that home exergaming is safe for this population in general. Minor technical issues, including sensor detection problems (particularly with seated exercises and use of walking frames) and connectivity challenges, were reported but were resolved within 24–48 h, with the program paused for the affected participant until the issue was resolved, and did not result in participant harm.

## Discussion

This multi-center single-arm pilot study examined the feasibility and acceptability of caregiver-supported gamified telerehabilitation using the EvolvRehab platform for community-dwelling adults with functional limitations in Singapore, and explored preliminary functional outcome signals. Because the study had no control group, the within-group changes observed cannot be attributed to the intervention; the findings should be read as feasibility and acceptability evidence together with preliminary efficacy signals that require confirmation in controlled trials. With that caveat, the results suggest that this approach is worth evaluating further as a way to address persistent challenges in community rehabilitation service delivery.

### Feasibility and acceptability

The high compliance rate of 80.6% observed in this study compares favorably with adherence rates reported for conventional community rehabilitation programs, which typically range from 28 to 50% [3, 16]. This finding aligns with systematic review evidence suggesting that gamification elements can enhance motivation and engagement in rehabilitation [11]. The variety of games offered (4–8 per participant) likely contributed to sustained interest, as noted in clinician feedback emphasizing the importance of program variety to maintain engagement.

The mean exercise duration of approximately 24 min daily exceeds the minimum recommended dosage for therapeutic benefit. Research suggests that higher doses of exergaming (above 134 min per week) are associated with greater improvements in balance outcomes [13]. Our participants averaged approximately 168 min per week, placing them in this higher-benefit range and potentially contributing to the observed functional improvements.

The System Usability Scale score of 67.75 falls within the “marginally acceptable” range according to established interpretation guidelines [14, 17]. While this indicates room for improvement in system design, it is comparable to other telerehabilitation systems evaluated in similar populations. A recent study of a telerehabilitation system for patients with chronic conditions reported a median SUS score of 77.5 [18], while a fall prevention system achieved a score of 62 in older adults [19]. The variability in individual scores (range 42.5–80) suggests that usability is influenced by participant characteristics such as technological familiarity, cognitive status, and physical impairment level.

Notably, participants expressed reluctance to pay for telerehabilitation services at the cost of face-to-face sessions. This finding echoes clinician feedback about the importance of positioning telerehabilitation as a complement to, rather than replacement for, conventional therapy. Studies have shown that hybrid models combining in-person and remote care may achieve superior outcomes and higher acceptability than either modality alone [1].

### Functional outcome signals

The within-group changes observed across several functional domains are consistent with the possibility of a therapeutic benefit from gamified telerehabilitation, but, in the absence of a control group, they cannot be interpreted as effects of the intervention and may also reflect natural recovery, concurrent conventional rehabilitation, or regression to the mean. The MBI change of 5.4 points from baseline to follow-up corresponds to a modest within-group gain in daily living activities. The SPPB change of 1.0 point post-intervention is of a magnitude comparable to the minimal clinically important difference for the SPPB, which Perera et al. (2006) reported as approximately 1.0 point for this measure [20], though a recent study suggested an MCID of 3 points in older adults with cardiovascular disease [21].

The 4.7-point within-group change in Berg Balance Scale scores from baseline to follow-up approaches the MCID of 5 points established for stroke patients requiring walking assistance [15]. This pattern is in the same direction as systematic review evidence on exergaming for balance training in older adults [13, 22]. The games’ focus on reaching, stepping, and trunk control directly target balance-related motor skills, potentially explaining the observed improvements.

At the 10-week follow-up after device retrieval, within-group values for MBI, BBS, and grip strength remained above baseline, whereas the SPPB improvement seen at the post-intervention assessment was not maintained relative to baseline. The pattern for the measures that remained elevated is compatible with the development of exercise habits that persisted beyond the formal intervention period, and participants self-reported increased willingness to continue exercising independently; however, without a control group these observations cannot be attributed to the intervention, and the non-sustained SPPB result indicates that not all gains were durable.

### Safety considerations

The absence of adverse events in this study is encouraging and is consistent with systematic review evidence on the generally safe profile of telerehabilitation, but it should be interpreted cautiously. The favourable safety record was obtained in a small, selected sample studied under caregiver-supported conditions, with competency-checked caregivers present at every session, therapist-guided progression, weekly tolerance review, and exclusion of higher-risk participants who lacked an adequate caregiver. The finding therefore applies to this specific, supported context and should not be generalised to unsupervised home exergaming in this population [2]. The caregiver competency assessment and requirement for caregiver presence during sessions likely contributed to safety. Previous studies using the EvolvRehab system similarly reported no falls across hundreds of successfully completed sessions [12].

### Implementation considerations

Clinician feedback highlighted several implementation considerations for future deployment. Recruitment challenges arose from strict eligibility criteria, with many potential participants excluded due to advanced age (> 90 years), cognitive impairment, or high assistance requirements. Future programs may need adapted criteria or modified interventions to reach broader populations. Technical issues including sensor detection problems (particularly for seated exercises and patients using mobility aids) required troubleshooting, underscoring the importance of robust technical support infrastructure. Beyond these points, the feasibility demonstrated here should be understood in the context of a relatively intensive support model. Each participant required up to three onboarding sessions, structured caregiver competency training, in-home installation and later retrieval of the device, individualised exercise prescription and progression, weekly video consultations, ongoing remote monitoring, and technical troubleshooting when problems arose. This represents a substantial human and technical resource commitment per participant, and the program also depended on the availability of a willing and capable primary caregiver for every session. These requirements are themselves an important implementation limitation: the model as tested may be difficult to scale to routine clinical use without dedicated staffing, reimbursement mechanisms, and a reliable technical support pathway, and it may be less feasible for participants who do not have a suitable caregiver. Future work should quantify the staff time and cost per participant and explore lighter-touch support models, so that the trade-off between the intensity of support and the feasibility, safety, and outcomes of the program can be assessed explicitly.

The multi-site implementation revealed site-specific factors affecting recruitment and outcomes. HWA demonstrated the fastest recruitment, having integrated the system into their service offerings, while HNF served home-based therapy patients with different logistical considerations. This heterogeneity reflects real-world implementation challenges and suggests that telerehabilitation programs may need adaptation for different service delivery contexts.

### Limitations

This study has several limitations that should be acknowledged. First, the small sample size (*N* = 10) limits statistical power and generalizability of findings. The pilot nature of the study precludes definitive conclusions about effectiveness, and results should be interpreted as preliminary evidence requiring confirmation in larger trials. Second, the absence of a control group prevents attribution of improvements specifically to the telerehabilitation intervention; observed gains may partly reflect natural recovery, continued conventional rehabilitation, or regression to the mean. Third, the heterogeneous participant population (multiple diagnoses and varying impairment levels) introduces variability that may obscure diagnosis-specific effects. Fourth, potential selection bias may have resulted from the requirement for caregiver presence and adequate home space, potentially excluding more isolated or impaired individuals who are generally more dependent. Finally, the short follow-up period of 10 weeks post-intervention limits understanding of long-term sustainability of effects. Several of these limitations warrant fuller emphasis. All participants continued to receive conventional outpatient rehabilitation during the study period; this concurrent care is a major uncontrolled confounder, and the within-group changes reported here cannot be separated from the effect of usual rehabilitation. In addition, the sample combined neurological (40%) and musculoskeletal (40%) conditions, which differ in their expected recovery trajectories and response to treatment. These diagnostic groups were pooled in the analysis without stratification, which limits interpretability, and the study was not powered to examine diagnosis-specific effects. The patient-reported acceptability data were collected with a custom questionnaire that has not been validated, so those findings are exploratory only. Finally, ten pairwise comparisons were conducted; although the false discovery rate correction was applied, the risk of spurious findings in a sample of this size remains, and the results should be read with that in mind. A future adequately powered randomised controlled trial with diagnosis-specific strata, predefined primary outcomes, validated acceptability instruments, and a control group would be needed to address these issues.

### Future directions

Future research should address the limitations of this pilot study through larger randomized controlled trials comparing gamified telerehabilitation to conventional rehabilitation or usual care. Diagnosis-specific studies focusing on populations such as stroke survivors, individuals with Parkinson’s disease, or older adults at risk of falls would help establish optimal target populations. Cost-effectiveness analyses are needed to inform policy decisions about integration of telerehabilitation into healthcare systems. Usability improvements based on participant feedback should be implemented and evaluated, potentially including voice control options, simplified interfaces, and improved seated exercise detection. Finally, hybrid models combining telerehabilitation with periodic in-person sessions warrant investigation as potentially optimal service delivery approaches.

## Conclusions

This multi-center single-arm pilot study indicates that caregiver-supported gamified telerehabilitation using the EvolvRehab platform is feasible and broadly acceptable for community-dwelling adults with functional limitations, and was delivered without adverse events under caregiver-supported conditions. High compliance (80.6%) and the within-group functional outcome signals observed for MBI, BBS, and grip strength are encouraging; however, the improvement in SPPB was not sustained at follow-up, and, because the study had no control group, none of these changes can be attributed to the intervention. The findings should therefore be read as feasibility and acceptability evidence together with preliminary efficacy signals, not as evidence of effectiveness. System usability requires refinement, the resource and caregiver burden of the support model needs to be addressed for broader adoption, and the cost concerns raised by participants remain unresolved. With these caveats, the study provides a reasonable basis for a larger, adequately powered randomised controlled trial that would be needed to determine whether gamified telerehabilitation can improve functional outcomes and extend rehabilitation services into home settings.

## Data Availability

Data available upon reasonable request to the corresponding author.

## References

[1] Jaswal S, Lo J, Howe A, Hao Y, Zhu S, Sithamparanathan G, et al. The Era of Technology in Healthcare—An Evaluation of Telerehabilitation on Client Outcomes: A Systematic Review and Meta-analysis. J Occup Rehabil. 2025;35(4):783–99. 10.1007/s10926-024-10237-4. 39340733 10.1007/s10926-024-10237-4 PMC12575596

[2] Shnitzer H, Chan J, Yau T, McIntyre M, Andreoli A, Kua A, et al. The Safety of Telerehabilitation: Systematic Review. JMIR Rehabil Assist Technol. 2025;12:e68681. 10.2196/68681. 40632682 10.2196/68681 PMC12266302

[3] Koh GC, Saxena SK, Ng TP, Yong D, Fong NP. Effect of duration, participation rate, and supervision during community rehabilitation on functional outcomes in the first poststroke year in Singapore. Arch Phys Med Rehabil. 2012;93(2):279–86. 10.1016/j.apmr.2011.08.017. 22289238 10.1016/j.apmr.2011.08.017

[4] Chen AW, Koh YT, Leong SW, Ng LW, Lee PS, Koh GC. Post community hospital discharge rehabilitation attendance: Self-perceived barriers and participation over time. Ann Acad Med Singap. 2014;43(3):136–44. 24714707

[5] Lui SK, Nguyen MH. Elderly Stroke Rehabilitation: Overcoming the Complications and Its Associated Challenges. Curr Gerontol Geriatr Res. 2018;2018:9853837. 10.1155/2018/9853837. 30050573 10.1155/2018/9853837 PMC6040254

[6] Essery R, Geraghty AW, Kirby S, Yardley L. Predictors of adherence to home-based physical therapies: a systematic review. Disabil Rehabil. 2017;39(6):519–34. 10.3109/09638288.2016.1153160. 27097761 10.3109/09638288.2016.1153160

[7] Alwadai B, Lazem H, Almoajil H, Hall AJ, Mansoubi M, Dawes H. Telerehabilitation and Its Impact Following Stroke: An Umbrella Review of Systematic Reviews. J Clin Med. 2024;14(1). 10.3390/jcm14010050. 10.3390/jcm14010050 PMC11721391 39797132

[8] Thanakamchokchai J, Khobkhun F, Phetsitong R, Chaiyawat P, Areerak K, Niemrungruang K, et al. Effectiveness of telerehabilitation on the International Classification of Functioning, Disability, and Health framework outcomes during the COVID-19 pandemic: A systematic review and meta-analysis of randomized controlled trials. Digit Health. 2025;11:20552076251325993. 10.1177/20552076251325993. 40162161 10.1177/20552076251325993 PMC11951915

[9] Dias JF, Oliveira VC, Borges PRT, Dutra F, Mancini MC, Kirkwood RN, et al. Effectiveness of exercises by telerehabilitation on pain, physical function and quality of life in people with physical disabilities: a systematic review of randomised controlled trials with GRADE recommendations. Br J Sports Med. 2021;55(3):155–62. 10.1136/bjsports-2019-101375. 33060156 10.1136/bjsports-2019-101375

[10] Molina-Garcia P, Mora-Traverso M, Prieto-Moreno R, Díaz-Vásquez A, Antony B, Ariza-Vega P. Effectiveness and cost-effectiveness of telerehabilitation for musculoskeletal disorders: A systematic review and meta-analysis. Ann Phys Rehabil Med. 2024;67(1):101791. 10.1016/j.rehab.2023.101791. 38128150 10.1016/j.rehab.2023.101791

[11] Rytterström P, Strömberg A, Jaarsma T, Klompstra L. Exergaming to Increase Physical Activity in Older Adults: Feasibility and Practical Implications. Curr Heart Fail Rep. 2024;21(4):439–59. 10.1007/s11897-024-00675-9. 39023808 10.1007/s11897-024-00675-9 PMC11333506

[12] Ren I, Rozanski G, Fernandez N, Zabala A, Ramos A, Arrinda I, et al. Exergaming delivery of a balance and fall prevention program for older adults: A feasibility study. Digit Health. 2022;8:20552076221144105. 10.1177/20552076221144105. 36569823 10.1177/20552076221144105 PMC9772941

[13] Cieślik B, Mazurek J, Wrzeciono A, Maistrello L, Szczepańska-Gieracha J, Conte P, et al. Examining technology-assisted rehabilitation for older adults’ functional mobility: a network meta-analysis on efficacy and acceptability. npj Digit Med. 2023;6(1):159. 10.1038/s41746-023-00907-7. 37620411 10.1038/s41746-023-00907-7 PMC10449892

[14] Bangor A, Kortum P, Miller TP. The System Usability Scale (SUS): an Empirical evaluation. Int J Hum Comput Interact. 2008;24:574. 10.1080/10447310802205776.

[15] Tamura S, Miyata K, Kobayashi S, Takeda R, Iwamoto H. The minimal clinically important difference in Berg Balance Scale scores among patients with early subacute stroke: a multicenter, retrospective, observational study. Top Stroke Rehabil. 2022;29(6):423–9. 10.1080/10749357.2021.1943800. 34169808 10.1080/10749357.2021.1943800

[16] Levy T, Laver K, Killington M, Lannin N, Crotty M. A systematic review of measures of adherence to physical exercise recommendations in people with stroke. Clin Rehabil. 2019;33(3):535–45. 10.1177/0269215518811903. 30458647 10.1177/0269215518811903 PMC6416703

[17] Lewis JR, Sauro J. Quantifying the User Experience: Practical Statistics for User Research. 2nd ed. Cambridge, MA: Morgan Kaufmann; 2016.

[18] Rossetto F, Borgnis F, Isernia S, Foglia E, Garagiola E, Realdon O, et al. System Integrated Digital Empowering and teleRehabilitation to promote patient Activation and well-Being in chronic disabilities: A usability and acceptability study. Front Public Health. 2023;11:1154481. 10.3389/fpubh.2023.1154481. 37250091 10.3389/fpubh.2023.1154481 PMC10214955

[19] Vaziri DD, Aal K, Ogonowski C, Von Rekowski T, Kroll M, Marston HR, et al. Exploring user experience and technology acceptance for a fall prevention system: results from a randomized clinical trial and a living lab. Eur Rev Aging Phys Activity. 2016;13(1):6. 10.1186/s11556-016-0165-z. 10.1186/s11556-016-0165-z PMC4902990 27293489

[20] Perera S, Mody SH, Woodman RC, Studenski SA. Meaningful change and responsiveness in common physical performance measures in older adults. J Am Geriatr Soc. 2006;54(5):743–9. 10.1111/j.1532-5415.2006.00701.x. 16696738 10.1111/j.1532-5415.2006.00701.x

[21] Tamura S, Miyata K, Igarashi T, Iizuka T, Otani T, Usuda S. Minimal clinically important difference of the short physical performance battery and comfortable walking speed in old-old adults with acute cardiovascular disease: a multicenter, prospective, observational study. Disabil Rehabil. 2023;45(6):1079–86. 10.1080/09638288.2022.2052978. 35341435 10.1080/09638288.2022.2052978

[22] Stanmore E, Eost-Telling C, Meekes W, Banham K, Chillala J, Roy B, et al. Exergames for falls prevention in sheltered homes: a feasibility study. Front Public Health. 2024;12:1344019. 10.3389/fpubh.2024.1344019. 38975352 10.3389/fpubh.2024.1344019 PMC11227257

